# Infertility risk assessment with ultrasound in congenital adrenal hyperplasia male patients

**DOI:** 10.1038/s41598-024-62954-8

**Published:** 2024-05-27

**Authors:** Zhiqian Wang, Ronghui Wang, Xing Wang, Sichang Zheng, Min Li, Yifei Yu, Zhenhua Liu, Shouyue Sun, Weiwei Zhan

**Affiliations:** 1 Department of Ultrasound, Ruijin Hospital, Shanghai Jiao Tong University School of Medicine, 2nd Ruijin Road 197, Shanghai, 200025 People’s Republic of China; 2 Department of Ultrasound, Ruijin-Hainan Hospital Shanghai Jiao Tong University School of Medicine Hainan Boao Research Hospital, Hainan, 571437 People’s Republic of China; 3 Department of Endocrine and Metabolic Diseases, Shanghai Institute of Endocrine and Metabolic Diseases, Ruijin Hospital, Shanghai Jiao Tong University School of Medicine, 2nd Ruijin Road 197, Shanghai, 200025 People’s Republic of China

**Keywords:** Urogenital diseases, Adrenal gland diseases, Endocrine reproductive disorders, Endocrine reproductive disorders

## Abstract

Testicular adrenal rest tumor (TART) is a prevalent complication associated with congenital adrenal hyperplasia (CAH), culminating in gonadal dysfunction and infertility. Early hormonal intervention is preventive, but excessive glucocorticoid poses risks. Developing reliable methods for early TART diagnosis and monitoring is crucial. The present study aims to formulate a scoring system to identify high-risk infertility through analysis of TART ultrasound features. Grayscale and power Doppler ultrasound were employed in this retrospective study to evaluate testicular lesions in male CAH patients. Lesion assessment encompassed parameters such as range, echogenicity, and blood flow, and these were subsequently correlated with semen parameters. Results of 49 semen analyzes from 35 patients demonstrated a notable inverse correlation between lesion scores and both sperm concentration (r_s_ = − 0.83, *P* < 0.001) and progressive motility (r_s_ = − 0.56,* P* < 0.001). The ROC curve areas for evaluating oligospermia and asthenozoospermia were calculated as 0.94 and 0.72, respectively. Establishing a lesion score threshold of 6 revealed a sensitivity of 75.00% and specificity of 93.94% for oligospermia and a sensitivity of 53.85% and specificity of 100.00% for asthenozoospermia. These findings underscore the potential utility of incorporating ultrasound into routine CAH patient management, facilitating timely interventions to preserve male fertility.

## Introduction

Congenital adrenal hyperplasia (CAH) is a rare autosomal recessive disorder that may cause testicular involvement in 40% of patients^1^. The presence of testicular involvement may result in the compression of the seminiferous tubules, causing irreversible damage to the surrounding testicular tissue. Eventually, it may lead to gonadal dysfunction and infertility^2^. While early hormonal intervention can avoid damage to gonadal function^3^, excessive use of glucocorticoids to inhibit ACTH production can lead to serious hormone-related side effects involving musculoskeletal, gastrointestinal, cardiovascular, endocrine, neuropsychiatric, dermatological, ocular, and immune systems^4,5^. Thus, it is important to develop methods for early diagnosis and monitoring of testicular adrenal rest tumors (TART).

Ultrasound (US) is a non-ionizing imaging technique that preserves the functionality of the testis. According to the clinical practice guideline of the Endocrine Society, US is recommended for assessing TART in men with CAH^6^. Although both US and magnetic resonance imaging (MRI) are equally effective in visualizing TART lesions, US is preferred as the initial diagnostic modality due to its lower cost, convenience, and ability to promptly identify patients at a higher risk of infertility^7^. This early detection enables timely referral for surgical intervention or sperm cryopreservation, thus preserving fertility by preventing irreversible damage.

In this study, we aim to develop a scoring system for high-risk infertility identification via analysis of the ultrasound features of TART ultrasound.

## Methods

### Participants

Medical records of CAH male patients treated in our hospital from November 2014 to March 2024, were retrospectively reviewed. Each testis was considered as an individual research object. A clinical diagnosis of TART in CAH patients with testicular involvement was established when the testicular lesions showed a decrease or disappearance following the administration of glucocorticoids. Patients whose lesions did not respond to hormone therapy were referred to urologists to rule out the presence of other testicular neoplasms, as recommended by the Endocrine Society^6^.

The exclusion criteria was presented as follows:

1. Incomplete clinical data including the absence of hormone tests and ultrasound images;
2. Chromosomal abnormalities;
3. Ultrasound-detected imaging findings potentially impacting semen quality, including microlithiasis^8^, heterogeneous echogenicity of the testicles^9^, significant testicular atrophy (testicular volume ≤ 5 mL), and epididymal obstruction;
4. Decreased level of inhibin B (INH B) or elevated level of follicle-stimulating hormone (FSH) in patients without testicular lesions.

For patients without testicular involvement, a decreased level of INH B or an elevated level of FSH are considered indicators of primary spermatogenic dysfunction^10,11^. Thus, participants with above hormonal abnormalities were excluded from this study. All the enrolled patients in this study were performed US after the administration of glucocorticoids. This practice stems from the hereditary nature of CAH, often leading to diagnoses at birth for a significant portion of patients.

### Study protocol

B-mode and power Doppler (PD) images of the testes were analyzed in axial and transverse sections for this study. In addition, levels of 17-hydroxyprogesterone (17-OHP), androstenedione (AD), adreno-cortico-tropic hormone (ACTH), and testosterone (T) were collected. Semen examination results including measurements of sperm concentration and progressive motility are also collected. On the same day as the hormone level tests and semen analyses, an US examination of the testicle was performed. All the aforementioned data were recorded for subsequent analysis.

### Ultrasound

The US system used in this study was the Esaote Mylab 65, equipped with a superficial linear array probe LA523 (4–13 MHz), for scanning the scrotum. For each case, the B-mode settings were adjusted to the highest transmit frequency that provided a complete display of the lesion and surrounding tissue. Additionally, the power Doppler gain was increased until the appearance of artifactual Doppler signals, and then decreased to minimize spurious signals. The power Doppler parameters were as follows: frequency 6.3 MHz, pulse repetition frequency (PRF) 500 Hz, and wall filter 3. Trans-scrotal US examinations were performed by one of three experienced sonographers. Using the Prader orchidometer formula, the ultrasound measurements of testicular volume were calculated as follows: length (L) × width (W) × height (H) × 0.71^12^. The recorded US images were independently interpreted by two radiologists (ZQ.W and RH.W) who possessed over 8 years of experience in andrological ultrasound. In case of any discrepancies, the third radiologist (ZH.L) with 12 years of experience was consulted for resolution. All reviewers were blinded to the written reports and laboratory test results. Given that TART predominately affects the testicular interstitum^13^, indicators of range, echogenicity, and vascularity, reflecting the degree of interstitial fibrosis, were adopt to develop a scoring system. The scoring criteria are provided in Table 1. The total score for each examination is the average of the scores assigned to bilateral testicles, which is the sum of scores from the three parameters. It is important to note that calcifications unrelated to TART lesions, such as testicular microlithiasis, were not included in the scoring.

**Table 1:** Principle of TART lesions score.

US features	Score	Explanation
Range	1	Lesions are not detectable by scrotal ultrasound
	2	Aggregated oval lesions (one, or separate multiples) in the mediastinum, involving no more than 1/3 of the testis on both transverse and sagittal sections
	3	The lesion fills the mediastinum and does not involve more than 1/3 of the transverse and sagittal of the testis; or the lesion does not fill the mediastinum, but involves more than 1/3 of the transverse or sagittal of the testis
	4	Under the circumstance of all the mediastinal involvement, the lesion on the transverse involves more than 1/3 of the testis with residence of normal testicular tissue
	5	Diffusely growing lesions without normal testicular tissue
Echogenicity	1	Homogeneous hypoechogenicity
	2	Heterogeneous hyperechoic
	3	Heterogeneous hyperechoic with calcification
Vascularity	1	Abundant linear blood flow signals filling the entire lesion
	2	Moderate linear blood flow signals occupying part of the lesion
	3	None blood flow or very few around lesion

### Semen quality evaluation

Semen analysis (SA) was performed using the Computer-Assisted Semen Analysis (CASA). In accordance with the guidelines set by the American Urological Association (AUA) and the American Society for Reproductive Medicine (ASRM)^14^, the lower reference limit for sperm concentration was determined to be 15 × 10^6^/ml, and the lower reference limit for progressive motility was set at 32%.

### Hormone measurement

Blood samples were procured at 08:00 A.M. ACTH levels were ascertained through the ELSA-ACTH immunoradiometric assay (Cisbio Bioassays, Codolet). Basel levels of 17-OHP and AD were determined utilizing the radioimmunoassay (Beckman Coulter Corp). INH B, FSH, and T levels were measured via the chemiluminescence immunoassay (Abbott Laboratories, Abbott Park, IL).

### Sample size calculation

Due to the rarity of TART and the limited size of the population, it was not feasible to perform a sample size calculation for this study.

### Statistic

Kendall’s tau-b correlation analysis between ultrasound scores and hormone levels was conducted across all patients. Additionally, for patients with semen examination results, Spearman correlation test or Kendall’s tau-b correlation analyses were carried out to explore the relationships among ultrasound scores, semen quality parameters, and hormone levels.

Receiver operating characteristic (ROC) curves were plotted to evaluate the validity of the scoring system, and the area under the curve (AUC) was calculated. The threshold that provided the best balance between sensitivity and specificity was determined using Youden’s method. The reliability of the scoring system was evaluated using the weighted Kappa statistic. The consistency of the score assigned by the two radiologists, was analyzed by Cohen κ. All statistical calculations were performed using IBM SPSS Statistics 25 software (IBM Corp.). Scatter plots with rugs were created using OriginPro 2021 software (OriginLab, Northampton), and a jitter plot was generated using the R programming language. The ROC curve analysis was conducted using MedCalc software (Version 20.014, MedCalc Software Ltd). A significance level of* P* < 0.05 was considered statistically significant.

### Ethical approval

This retrospective study was conducted in accordance with the approval of the Ruijin Hospital Ethics Committee, Shanghai JiaoTong University School of Medicine (AF0406/14.0/2023-03-01). As an observational study, the written informed consent was waived by Ruijin Hospital Ethics Committee, Shanghai JiaoTong University School of Medicine. All experiments were performed in accordance with relevant named guidelines and regulations.

## Results

### Patient characteristics

Data were collected from 89 male CAH patients, 7 of 89 patients were excluded from the analysis due to insufficient clinical data. Among 82 CAH patients, 54.9% (45/82) were found to have testicular involvement, 84.4% (38/45) exhibited bilateral testis involvement, and 15.6% (7/45) had unilateral involvement. The median age of the patients at the time of their first ultrasonography was 22 years, with a range of 8 to 61 years (interquartile range 18.0, 35.0) (Table 2).

**Table 2:** Clinical and demographic characteristics of the study population.

Variables	Median (range)
Age, y*	22 (8–61)
BMI, kg/m^2^	25.26 (15.20–43.28)
Blood pressure	
Systolic blood pressure, mmHg	124 (82–173)
Diastolic blood pressure, mmHg	79 (50–128)
Hormones	
ACTH (pg/ml)	20.59 (4.7–2000.0)
17-OHP (ng/ml)	2.39(0.09–50.10)
AD (ng/ml)	0.58 (0.03–13.01)
T (ng/ml)	3.77 (0.07–20.04)

ACTH, adreno-cortico-tropic hormone; 17-OHP, 17-hydroxyprogesterone; AD, androstenedione; T, testosterone.

*Age at the first ultrasonography.

82 CAH patients having 168 ultrasound and hormone examinations were included for the correlation analyses with scores and hormone levels. 46/82 patients underwent a single ultrasound examination, involving a total of 92 testicles, while 35/82 patients underwent two or more ultrasound examinations, resulting in a cumulative count of 110 examinations involving 220 testicles. Additionally, 1/82 patient, who underwent 12 follow-up visits, underwent a right-sided orchiectomy to determine the pathology of the lesion prior to presentation at our center. Consequently, a total of 324 testicles were enrolled in the study.

Among the cohort of 82 patients examined, 24 individuals lacked available semen test results, 16 patients with ultrasound-detected imaging findings potentially impacting semen quality (9 testicular atrophy, 4 testicular microlithiasis, 2 heterogeneous testicular echogenicity, and 1 epididymal obstruction), 1 manifested a chromosomal abnormality, and 6 patients devoid of discernible testicular lesions with decreased INH B levels or elevated FSH levels were excluded. Finally, 35 patients with semen test results were included in the correlation analysis, with a total of 49 tests. Of these, 25 patients underwent a single examination, while 7 patients underwent two examinations, 2 patients underwent three examinations, and 1 patient underwent four examinations.

**Figure 1:**
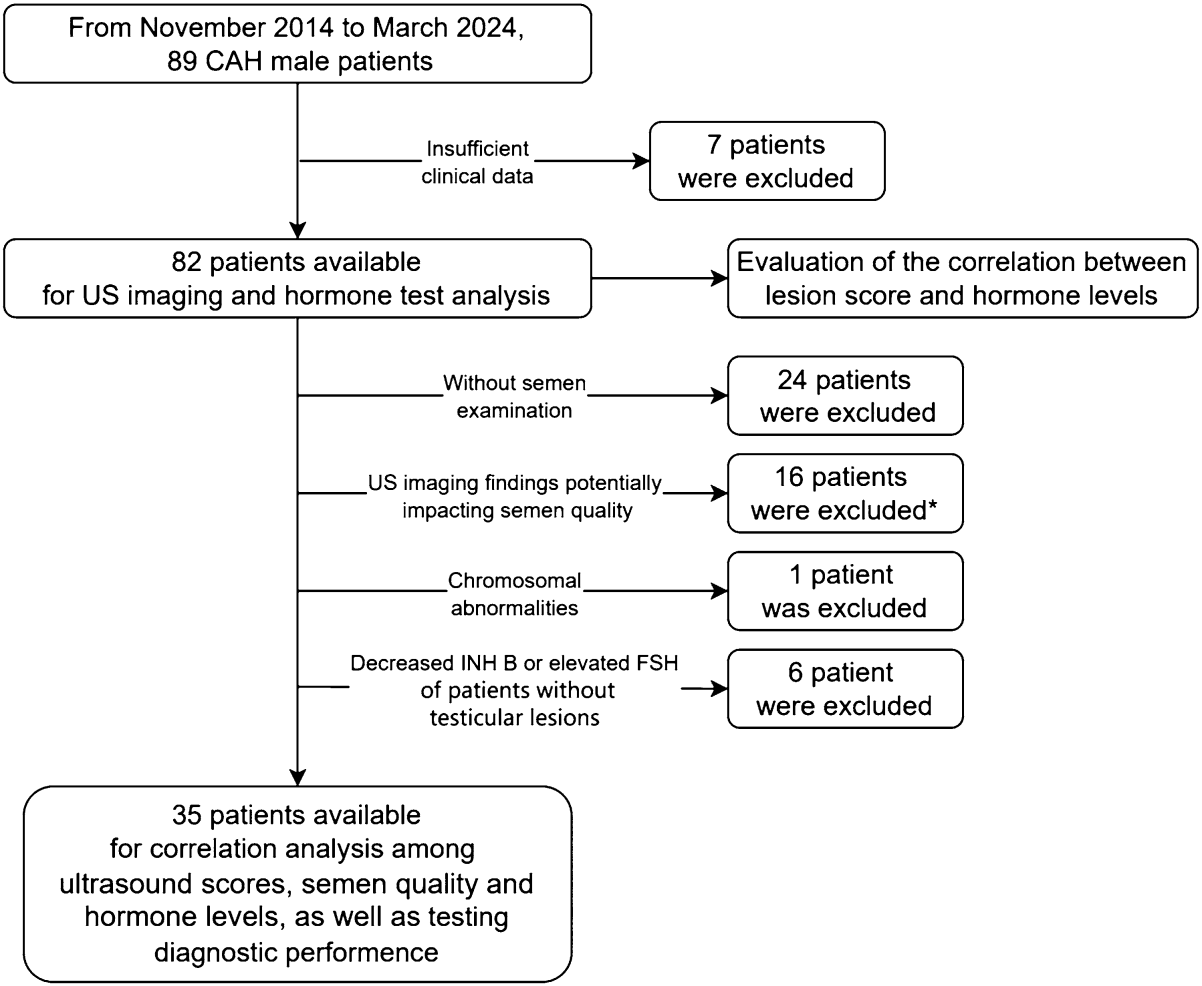
Flowchart of study population. *16 patients with US imaging findings potentially impacting semen quality included 9 significant testicular atrophy, 4 testicular microlithiasis, 2 heterogeneous echogenicity on US, and 1 epididymal obstruction.

**Figure 2:**
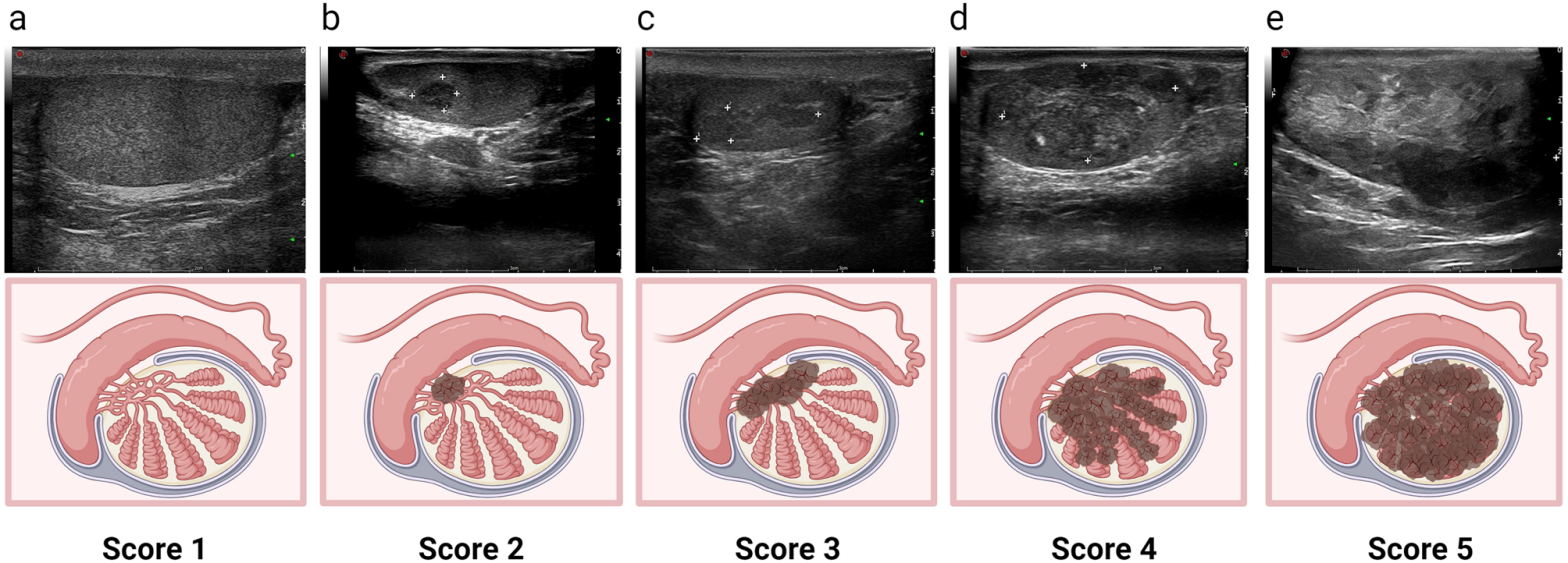
Typical images of testicular adrenal rest tumor (TART) involvement range scores. (**a**–**e**) show testicular B-mode ultrasound images of five male patients with congenital adrenal hyperplasia (CAH), aged 28, 41, 13, 11 and 17, respectively. (**a**) Score 1: Not detectable by scrotal ultrasound; (**b**) Score 2: Aggregated oval lesions in the mediastinum, involving no more than 1/3 of the sagittal section of the testis; (**c**) Score 3: The lesion fills the mediastinum and does not involve more than 1/3 of the transverse and sagittal of the testis; (**d**) Score 4: Under the circumstance of all the mediastinal involvement, the lesion on the transverse involves more than 1/3 of the testis with residence of normal testicular tissue; (**e**) Score 5: Diffusely growing lesion, no normal testicular tissue visible.

**Figure 3:**
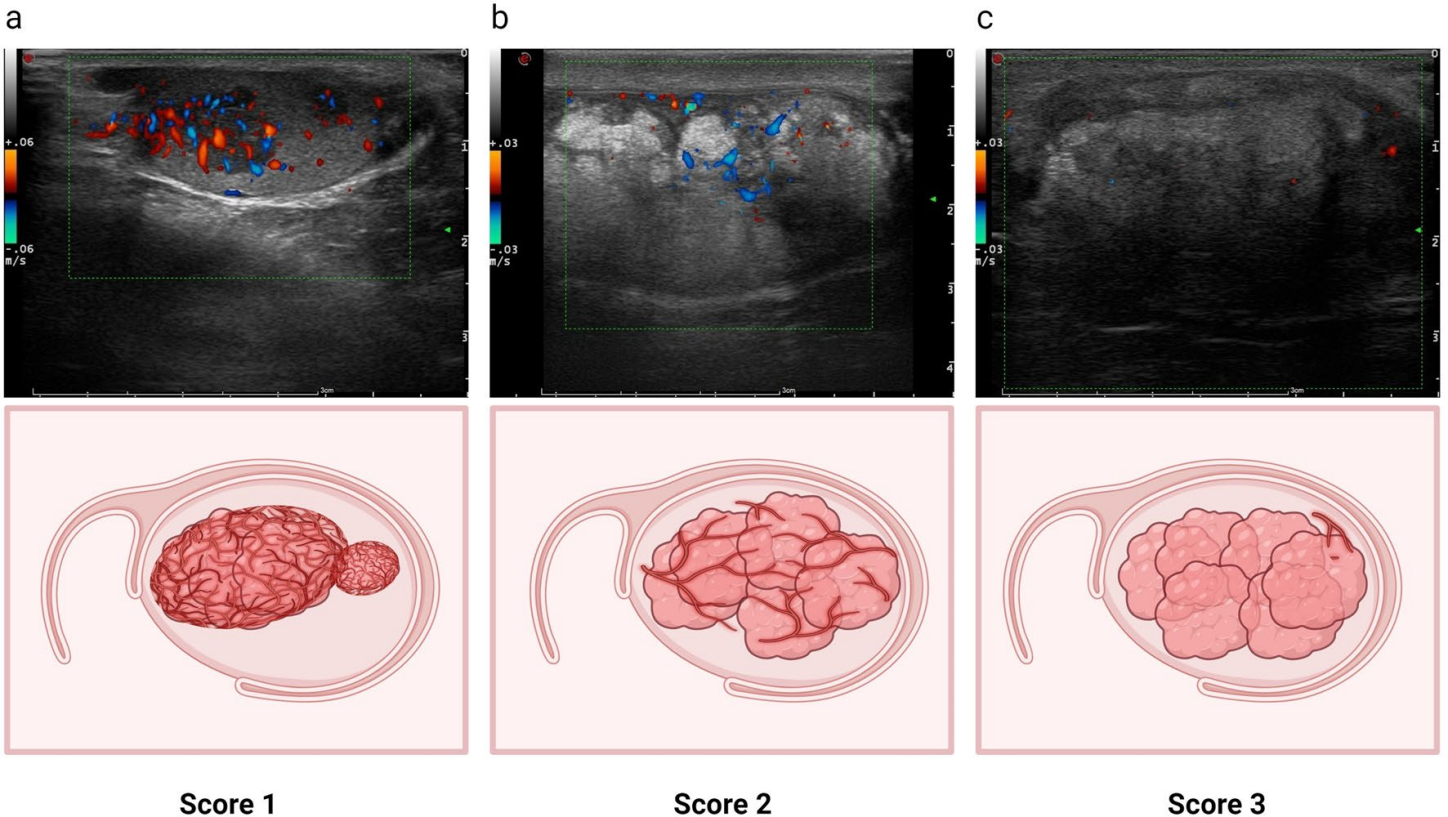
Typical images of testicular adrenal rest tumor (TART) vascularity patterns scores. (**a**–**c**) depict testicular power Doppler images of male CAH patients aged 13, 41, and 38, respectively. (**a**) Score1: Abundant linear blood flow signals filling the entire lesion; (**b**) Score 2: Moderate linear blood flow signals occupying part of the lesion; (**c**) Score 3: No blood flow or very limited blood flow around lesion.

The flowchart of study population was presented in Fig. 1. Figures 2 and Fig. 3 present representative images of B-mode and Doppler ultrasound with varying scores.

### Structure of scoring system

A significant correlation has been identified between the range, echogenicity, and vascularity scores, and the parameters pertaining to semen analysis. The Spearman correlation coefficients (r_s_) for sperm concentration were recorded as − 0.72 (*P* < 0.01), − 0.74 (*P* < 0.01), and − 0.74 (*P* < 0.01), respectively. Furthermore, the Spearman correlation coefficients for motility were observed to be − 0.35 (*P* < 0.05), − 0.37 (*P* < 0.05), and − 0.38 (*P* < 0.05) correspondingly. Thus, the assignment of the scoring system included the three categories of range, echogenicity, and vascularity scores.

### Diagnostic performance of US scoring system

**Figure 4:**
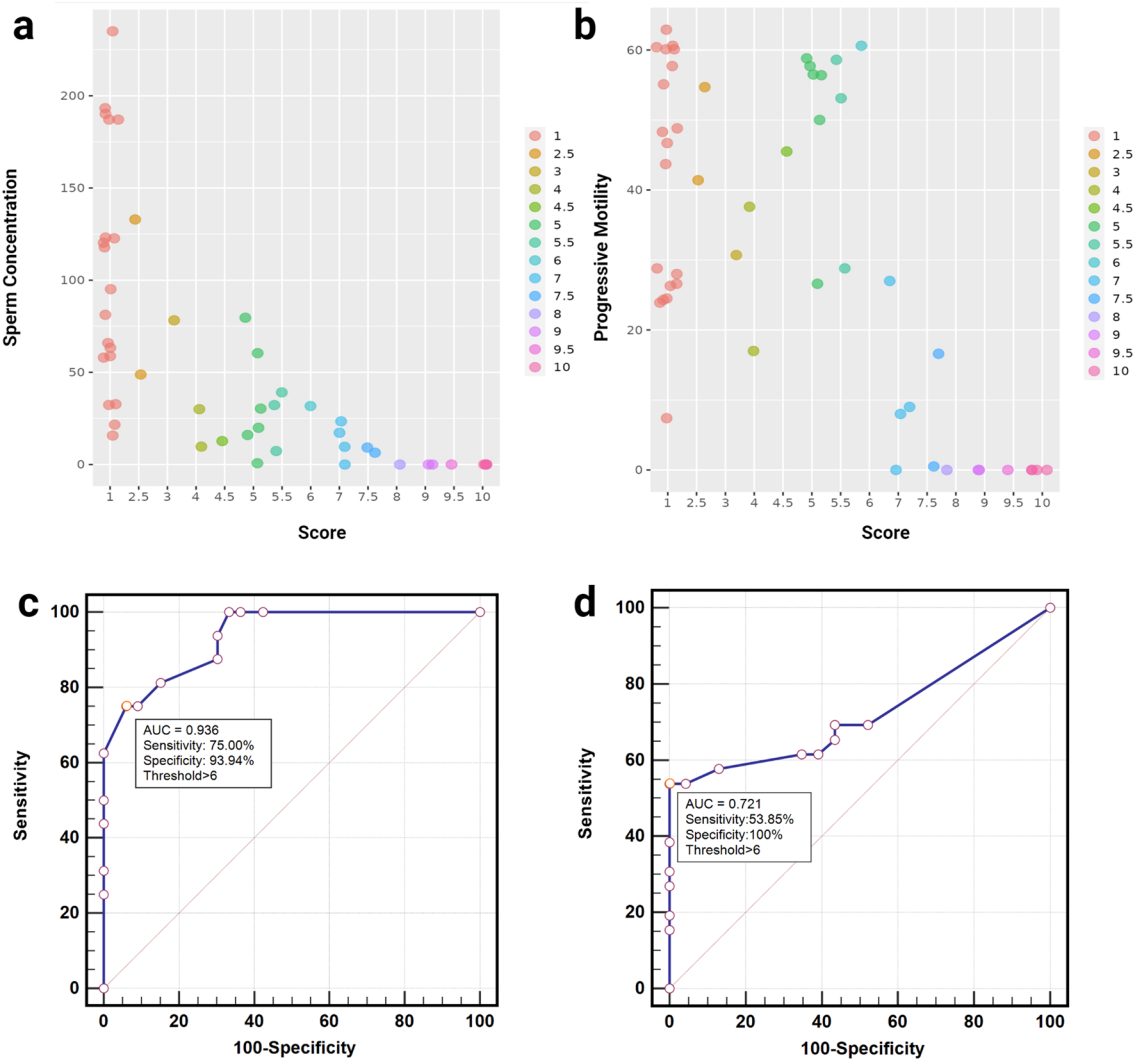
The jitter plots showing sperm (**a**) concentration and (**b**) progressive motility at different testicular adrenal rest tumor (TART) scores. Receiver operating characteristic (ROC) curves of oligospermia (**c**) and asthenospermia (**d**) based on consensus testicular adrenal rest tumor (TART) score.

Jitter plots demonstrated a negative correlation between sperm concentration and progressive motility with increasing TART lesion scores (Fig. 4). The Spearman correlation coefficients (r_s_) for sperm concentration and progressive motility were − 0.83 (*P* < 0.001) and − 0.56 (*P* < 0.001), respectively. The ROC analysis revealed that the AUC for the assessment of oligospermia based on the TART lesion score was 0.94 (95% CI 0.83, 0.99; *P* < 0.001), while the AUC for the assessment of asthenozoospermia was 0.72 (95% CI 0.57, 0.84; *P* = 0.003). A threshold value of 6 provided the best balance between sensitivity and specificity for both oligospermia and asthenozoospermia, yielding a sensitivity of 75.00% (95% CI 0.48, 0.93) and a specificity of 93.94% (95% CI 0.80, 0.99) for diagnosing oligospermia. For diagnosing asthenozoospermia, the threshold value of 6 resulted in a sensitivity of 53.85% (95% CI 0.33, 0.73) and a specificity of 100% (95% CI 0.85, 1.00) (Fig. 4). The relevant data of patients with multiple semen examinations have been listed in Supplemental data S.Table 2. In patients with scores less than 6, semen quality improved as the score decreased. However, in patients with scores greater than 6, semen quality did not improve significantly.

### Inter-observer consistency assessment

The two radiologists who assigned lesion scores demonstrated excellent agreement, as evidenced by a weighted kappa value (κ_w_) of 0.97(95%CI 0.96–0.99).

### Hormone evaluation

**Figure 5:**
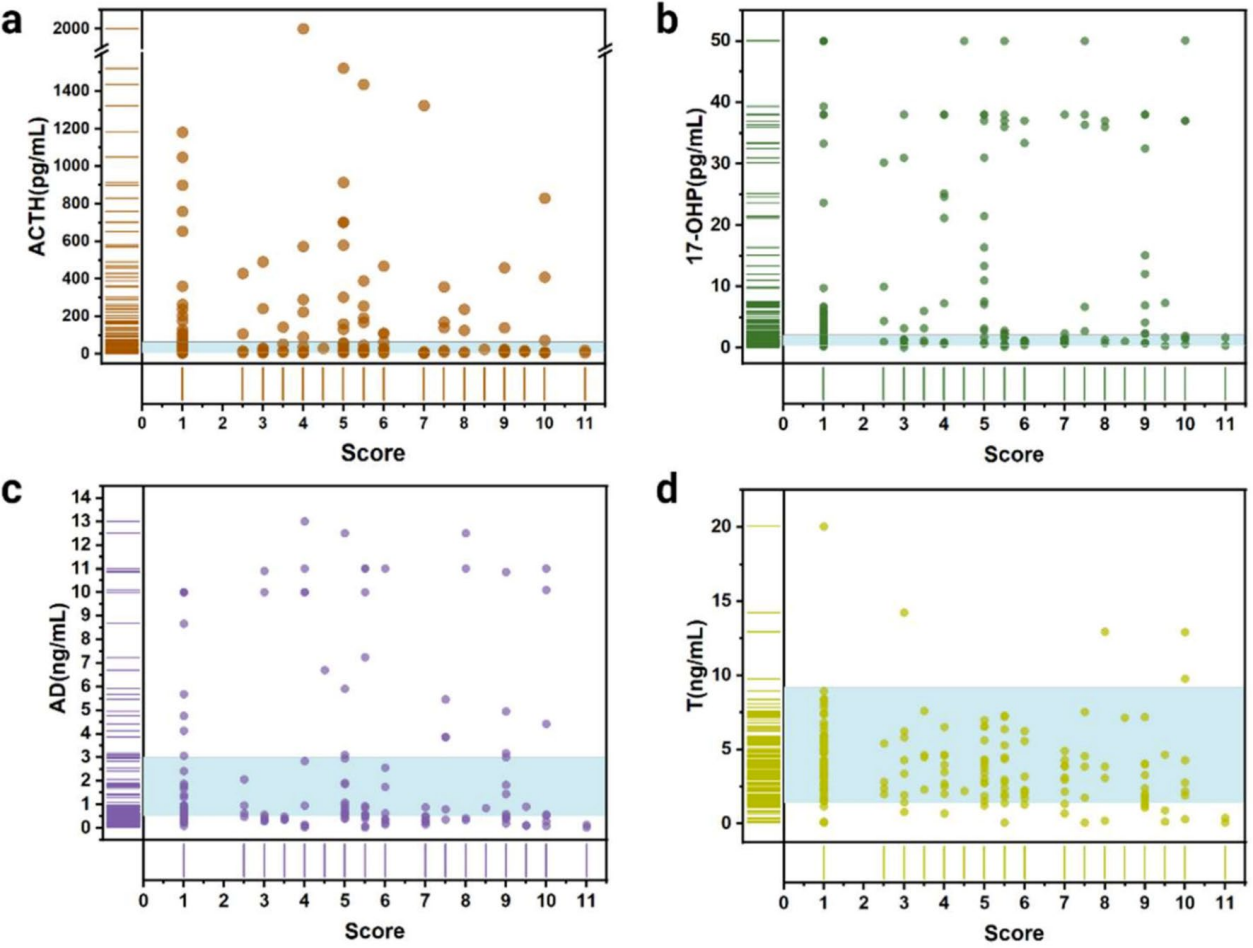
Scatter plots with rugs illustrating the levels of (**a**) adreno-cortico-tropic hormone (ACTH), (**b**) 17-hydroxyprogesterone (17-OHP), (**c**) androstenedione (AD), and (**d**) testosterone (T) at different testicular adrenal rest tumor (TART) scores. In the figure, the blue areas indicate the normal ranges.

Among all the analyses of hormonal levels, only testosterone (T) exhibited a weak negative correlation with increasing lesion scores (τ_b_ = − 0.19; *P* = 0.001), and a weak positive correlation with sperm concentration (τ_b_ = 0.24; *P* = 0.002) and progressive motility (τ_b_ = 0.18; *P* = 0.02). No statistically significant correlations were found between the lesion scores and the other hormone levels (Fig. 5). Additionally, there were no significant correlations observed between semen parameters and the hormone levels except T. The Kendall tau-b correlation coefficients and corresponding *p*-values are presented in Table 3.

**Table 3:** Kendall’s tau-b correlation coefficient among hormones, semen parameters and scores.

	ACTH	AD	17-OHP	T
SC^1^	− 0.09 (*P* = 0.23)	0.04(*P* = 0.58)	− 0.06(*P* = 0.44)	0.24(*P* = 0.002)**
PR^1^	− 0.05(*P* = 0.51)	− 0.02(*P* = 0.85)	− 0.10(*P* = 0.19)	0.18(*P* = 0.02)*
Score^2^	− 0.003(*P* = 0.95)	0.02(*P* = 0.76)	0.07(*P* = 0.21)	− 0.19(*P* = 0.001)**

^1^The number for analysis was 49 examinations from 35 patients.

^2^The number for analysis was 168 examinations from 82 patients.

Numbers in the spaces are the Kendall tau-b correlation coefficient, τ_b_.

SC, Sperm concentration; PR, Progressive motility.

*The correlation is significant at the *P* < 0.05 level. **The correlation is significant at the *P* < 0.01 level.

## Discussion

TART is a commonly observed condition in patients with CAH, and its occurrence cannot be prevented due to the negative feedback from impaired cortisol production, which allows hypersecreted ACTH to stimulate the proliferation of testicular adrenal remnant cells^15^. Claahsen-van der Grinten et al. described five distinct stages of the TART process based on histological features^2^: Stage (1) presence of adrenal rest cells in the rete testis; Stage (2) proliferation of adrenal rest cells; Stage (3) further growth of adrenal rest cells compressing the rete testis; Stage (4) Further hypertrophy and hyperplasia of adrenal rest cells leading to progressive obstruction of the rete testis may induce the initiation of fibrotic changes within the tumor and the occurrence of focal lymphocytic infiltration; Stage (5) finally, chronic obstruction resulting in destruction of surrounding testicular parenchyma and irreversible testicular damage.

In our current study, we found a similar range of US features among the included patients, which served as the basis for developing our scoring system. Our scoring system revealed a negative correlation between lesion score and sperm concentration (r_s_ = − 0.83, *P* < 0.001) as well as progressive motility (r_s_ = − 0.56, *P* < 0.001). Currently, the association between the size of TART lesion and ACTH levels remains controversial^16^. Our results showed that ACTH levels did not significantly correlate with the lesion scores (r_s_ = − 0.003, *P* = 0.95). We hypothesize that prolonged mechanical obstruction of the seminiferous tubules may lead to dysfunction of spermatogenic function and peritubular fibrosis^13^. Consequently, adrenal rest cells may dedifferentiate over time and lose their dependence on ACTH^2^, explaining the lack of correlation between ACTH and semen quality in TART patients.

Effective management of TART requires long-term approaches. Hormone therapy and surgical treatment are currently the main methods for preserving fertility in TART patients. Early-stage high-dose glucocorticoid treatment can reduce tumor size, prevent compression of the testicular rete, slow down tumor growth, and inhibit the development of testicular fibrosis, thereby preventing infertility^17^. However, in cases of longstanding TART, high-dose glucocorticoids may not always restore testicular spermatogenesis and can have side effects^18,19^. Testis-sparing surgery has been suggested to maximize fertility preservation in TART patients^19,20^. However, this procedure does not improve gonadal function in patients with longstanding TART^18,21,22^. Therefore, timely treatment before the irreversible loss of gonadal function is crucial. Our scoring system, based on readily available and easy-to-operate B-mode and power Doppler ultrasound, may serve as a basis for making surgical decisions before the onset of gonadal failure in TART patients. For oligospermia, a threshold of 6 yielded a sensitivity of 75.00% (95% CI 0.48, 0.93) and specificity of 93.94% (95% CI 0.80, 0.99) while for asthenospermia, a sensitivity of 53.85% (95% CI 0.33, 0.73) and specificity of 100% (95% CI 0.85, 1.00) were obtained. This study found that the semen quality of patients with scores greater than 6 did not change significantly over time, likely due to irreversible damage to testicular spermatogenic function. These findings demonstrate that the scoring system can serve as a valuable reference for decisions related to fertility preservation. Previous studies have indicated the desirability but costliness of sperm cryopreservation during the early stages of TART, necessitating a careful assessment of the benefit-risk balance^23^. Therefore, TART patients with scores exceeding 6 may have the risk of irreversible gonadal failure, attributed to a combination of obstructive and non-obstructive factors, whereby neither hormonal therapy nor surgery can enhance spermatogenic function. A timely sperm cryopreservation should be recommended.

Ultrasound, as a tool for morphological attribution in the assessment of male infertility, is widely known to be independent of environmental factors, which may be able to make up for the shortcomings of semen as a tool for testicular functional assessment, which is affected by the environment. In this study, the developed scoring system may serve as an alternative tool for screening high-risk male infertility in CAH. Additionally, it can guide patients in taking full advantage of relevant assisted reproductive methods to preserve their fertility. This is particularly important as TART can occur in children, and most fertility problems in CAH arise during childhood^24^. Although semen analysis is an important non-invasive method of monitoring reproductive function, it is not easy to obtain semen in prepubertal patients. During follow-up, US serves as an excellent imaging modality for evaluating TART, especially when intratesticular masses cannot be palpated. US enables the early detection of these masses, facilitating timely hormone replacement therapy and the prevention of potential infertility. Thus, ultrasonography fills the gap in evaluating gonadal function in children.

While CAH is rare, it typically manifests in early childhood and may lead to a shortened life expectancy, necessitating ongoing healthcare services. This condition often impacts the entire family, creating ongoing stress. Although TART is relatively uncommon, it is prevalent in CAH patients and can strain healthcare and social services disproportionately. Excessive hormone therapy solely to mitigate testicular lesions incurs unnecessary costs and risks significant side effects for patients. With timely diagnosis and proper treatment, these individuals can lead fulfilling lives and contribute positively to society.

## Limitations

TART, as a rare autosomal recessive disorder, the research sample size is limited. Meanwhile, as a retrospective study, this study absent the regular assessments conducted at consistent time intervals. To address these limitations and enhance the validity of the findings, further multicenter prospective studies involving larger sample sizes and regular follow-ups are warranted. Additionally, prospective studies are necessary to establish appropriate patient follow-up intervals over time in the future.

## Conclusions

To the best of our knowledge, this scoring system is first developed by our group, and there are no previous studies have reported on it. In conclusion, US can be used as the preferred imaging modality for the evaluation of TART. US should be included as a routine examination in the follow-up of patients with CAH to ensure early intervention before gonadal failure to preserve male fertility.

### Supplementary Information

Supplementary Table 1.

Supplementary Table 2.

## Data Availability

The datasets used and/or analysed during the current study are available from the corresponding author on reasonable request.
