# Supplementary material for: Infertility risk assessment with ultrasound in congenital adrenal hyperplasia male patients
Source: Sci Rep. 2024 May 27;14:12058. doi: 10.1038/s41598-024-62954-8 (PMC11130187; doi:10.1038/s41598-024-62954-8)
Supplement: Supplementary file 1 — Supplementary Table 1. [file 41598_2024_62954_MOESM1_ESM.docx]

**Supplemental Data**

**Supplemental Table S1. Precise data**

| **Patient No.** | **Age at diagnosis of CAH/Starting hormone therapy** | **Age at first ultrasound examination** | **Number of ultrasound examinations** | **Age at first semen test** | **Number of semen examinations** |
| --- | --- | --- | --- | --- | --- |
| **1** | 2 | 27 | 1 | 27 | 1 |
| **2** | 36 | 36 | 3 | 37 | 2 |
| **3** | 6 | 23 | 1 | 23 | 1 |
| **4** | 4 | 17 | 2 | 18 | 1 |
| **5** | 10 | 19 | 12 | 23 | 4 |
| **6** | 38 | 38 | 1 | 38 | 1 |
| **7** | 4 | 19 | 1 | 19 | 1 |
| **8** | 0 | 19 | 2 | 19 | 2 |
| **9** | 7 | 23 | 1 | 23 | 1 |
| **10** | 1 | 58 | 1 | 58 | 1 |
| **11** | 4 | 19 | 1 | 19 | 1 |
| **12** | 0 | 18 | 1 | 18 | 1 |
| **13** | 2 | 16 | 1 | 16 | 1 |
| **14** | 34 | 34 | 5 | 38 | 1 |
| **15** | 0 | 34 | 2 | 34 | 2 |
| **16** | 18 | 21 | 2 | 21 | 2 |
| **17** | 0 | 19 | 2 | 19 | 1 |
| **18** | 48 | 48 | 2 | 48 | 1 |
| **19** | 0 | 18 | 2 | 18 | 1 |
| **20** | 29 | 29 | 4 | 29 | 1 |
| **21** | 5 | 28 | 1 | 28 | 1 |
| **22** | 28 | 31 | 1 | 31 | 1 |
| **23** | 31 | 31 | 1 | 31 | 1 |
| **24** | 0 | 23 | 1 | 23 | 1 |
| **25** | 0 | 20 | 7 | 25 | 3 |
| **26** | 1 | 12 | 5 | 18 | 3 |
| **27** | 0 | 20 | 2 | 20 | 2 |
| **28** | 28 | 29 | 1 | 29 | 1 |
| **29** | 5 | 12 | 5 | 20 | 2 |
| **30** | 59 | 59 | 2 | 59 | 1 |
| **31** | 0 | 23 | 3 | 22 | 2 |
| **32** | 10 | 27 | 2 | 28 | 1 |
| **33** | 52 | 52 | 2 | 52 | 1 |
| **34** | 6 | 23 | 1 | 23 | 1 |
| **35** | 7 | 21 | 1 | 21 | 1 |
