# Supplementary material for: Infertility risk assessment with ultrasound in congenital adrenal hyperplasia male patients
Source: Sci Rep. 2024 May 27;14:12058. doi: 10.1038/s41598-024-62954-8 (PMC11130187; doi:10.1038/s41598-024-62954-8)
Supplement: Supplementary file 2 — Supplementary Table 2. [file 41598_2024_62954_MOESM2_ESM.docx]

**Supplemental Data**

**Supplemental Table S2.**

| **Patient No.** | **Number of semen tests** | **Data of semen tests**  **(YY-MM-DD)** | **US score of TART** | **Sperm concentration (10^6^/mL)** | **Progressive motility (%)** |
| --- | --- | --- | --- | --- | --- |
| 2 | 2 | 2021-12-20 | 4 | 9.7 | 37.6 |
|  |  | 2023-11-25 | 5.5 | 7.3 | 28.8 |
| 5 | 4 | 2021-11-08 | 10 | 0 | 0 |
|  |  | 2022-10-13 | 10 | 0 | 0 |
|  |  | 2023-05-08 | 10 | 0 | 0 |
|  |  | 2024-03-08 | 10 | 0 | 0 |
| 8 | 2 | 2023-06-06 | 1 | 123.1 | 24.3 |
|  |  | 2023-12-03 | 1 | 81.2 | 23.9 |
| 15 | 2 | 2021-12-03 | 5 | 30.3 | 26.6 |
|  |  | 2023-06-24 | 5.5 | 32.2 | 58.6 |
| 16 | 2 | 2023-05-08 | 1 | 21.6 | 60.4 |
|  |  | 2023-11-15 | 1 | 32.3 | 57.7 |
| 25 | 3 | 2021-07-19 | 4 | 30 | 17 |
|  |  | 2023-07-17 | 3 | 78.2 | 30.7 |
|  |  | 2024-02-14 | 2.5 | 132.9 | 54.7 |
| 26 | 3 | 2022-07-19 | 7 | 9.6 | 27 |
|  |  | 2022-12-06 | 7 | 23.4 | 8 |
|  |  | 2023-06-07 | 7 | 17.2 | 9 |
| 27 | 2 | 2021-01-22 | 9 | 0 | 0 |
|  |  | 2022-01-25 | 9.5 | 0 | 0 |
| 29 | 2 | 2023-07-27 | 1 | 190.2 | 48.8 |
|  |  | 2024-02-03 | 1 | 235 | 28.8 |
| 31 | 2 | 2023-02-09 | 5 | 79.6 | 57.7 |
|  |  | 2024-01-25 | 6 | 31.7 | 60.6 |
